# Spontaneous Bilateral Dissection of the Vertebral Artery: A Case Report

**DOI:** 10.7759/cureus.9310

**Published:** 2020-07-21

**Authors:** Mohammed F Almuaigel, Aldanah Althwanay, Abdullah Alamri

**Affiliations:** 1 Medical Education, College of Medicine, King Faisal University, Al-Ahsa, SAU; 2 Internal Medicine, Imam Abdulrahman Bin Faisal University, Khobar, SAU; 3 Neurology, Imam Abdulrahman Bin Faisal University, Khobar, SAU

**Keywords:** vertebral arteries, arterial dissection, stroke, basilar stroke, chiropractic, neck manipulation

## Abstract

Spontaneous dissection of the vertebral artery refers to cases that do not involve significant blunt or penetrating trauma as a precipitating factor. However, cases of spontaneous dissection of the vertebral artery do have a history of trivial or minor injury involving some degree of cervical distortion such as chiropractic neck manipulation, as the extreme hyperextension and/or rotation of the neck may create areas of stretch and lead to intimal or adventitial tears in the vertebral artery causing dissection. It is a relatively rare, potentially disabling and sometimes an under-diagnosed cause of stroke. It accounts for 2% of all ischemic strokes and 7% of the cases are bilateral. Herein we present a case of bilateral vertebral artery dissection complicated by basilar artery stroke in a young male patient following chiropractic manipulation of the neck. Neuroimaging modalities upon presentation confirmed the diagnosis. Antiplatelets were administered, and a great clinical outcome after three months was achieved. This report demonstrates the potential hazards associated with neck trauma, including chiropractic manipulation, as it is under reported in Saudi Arabia.

## Introduction

Spontaneous dissection of the vertebral artery (sVAD) is a relatively rare but increasingly recognized cause of stroke in patients younger than 45 years [1]. It accounts for 2% of all ischemic strokes, and 7% of the cases are bilateral. It is considered a potentially disabling, and probably an under-diagnosed cause of stroke in young adults. Although the term sVAD is used to describe cases that do not involve significant blunt or penetrating trauma as a precipitating factor, many patients with so-called sVAD have a history of trivial or minor injury involving some degree of cervical distortion such as chiropractic neck manipulation [1-3].

There are two vertebral arteries (VAs) both left and right, ascending vertically through the transverse foramina of the cervical vertebrae (C6-C1). The VAs join to form the basilar artery, primarily supplying the brainstem, cerebellum, and occipital lobes [4]. Extreme hyperextension and/or rotation of the neck may create areas of stretch and lead to intimal or adventitial tears in the vertebral artery, thereby causing dissection [5]. Hence, chiropractic is a potential risk factor for vertebral dissection [6]. Interestingly, chiropractic therapy is rare among the Saudi population with a prevalence of 4.1% of all the complementary therapy seeking Saudi population. Moreover, the majority of chiropractic therapy is performed by traditional healers instead of medical practitioners [7]. The presentation of symptoms such as neck pain and ischemic symptoms may be immediate or delayed days to weeks post-injury [6]. Search of the relevant literature did not reveal similar cases in Saudi Arabia; hence the true impact of chiropractic manipulation as a potential cause of stroke in the Saudi population cannot be determined.

## Case presentation

A 30-year-old male presented to the emergency room after experiencing sudden vertigo and difficulty standing. The patient reported frequent chiropractic manipulation of the neck in a local private center. On that day of presentation, and during neck manipulation, he experienced severe vertigo, weakness on the left side of the body and inability to stand unassisted. Two hours later, he noticed difficulty talking and swallowing. The patient denied any neurologic symptoms prior to neck manipulation or similar episodes in the past. He is a known smoker, but with no chronic diseases or other pertinent medical or family history. On physical examination, he had horizontal nystagmus, dysarthria, an absent gag reflex with difficulty swallowing, right sided facial dropping, and left hemiparesis.

**Figure 1:**
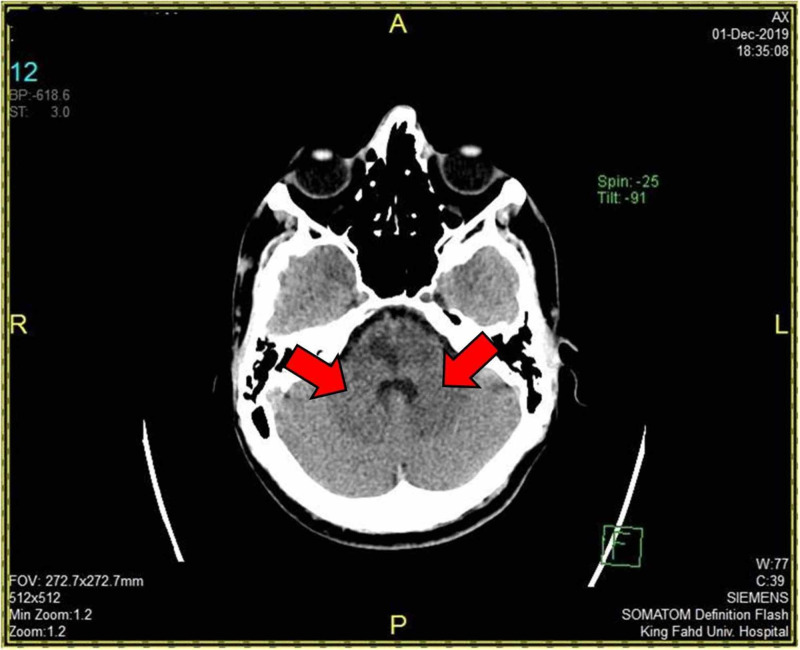
Computed tomography (CT) of the brain Axial view of the brain reveals pontine hypodensity.

**Figure 2:**
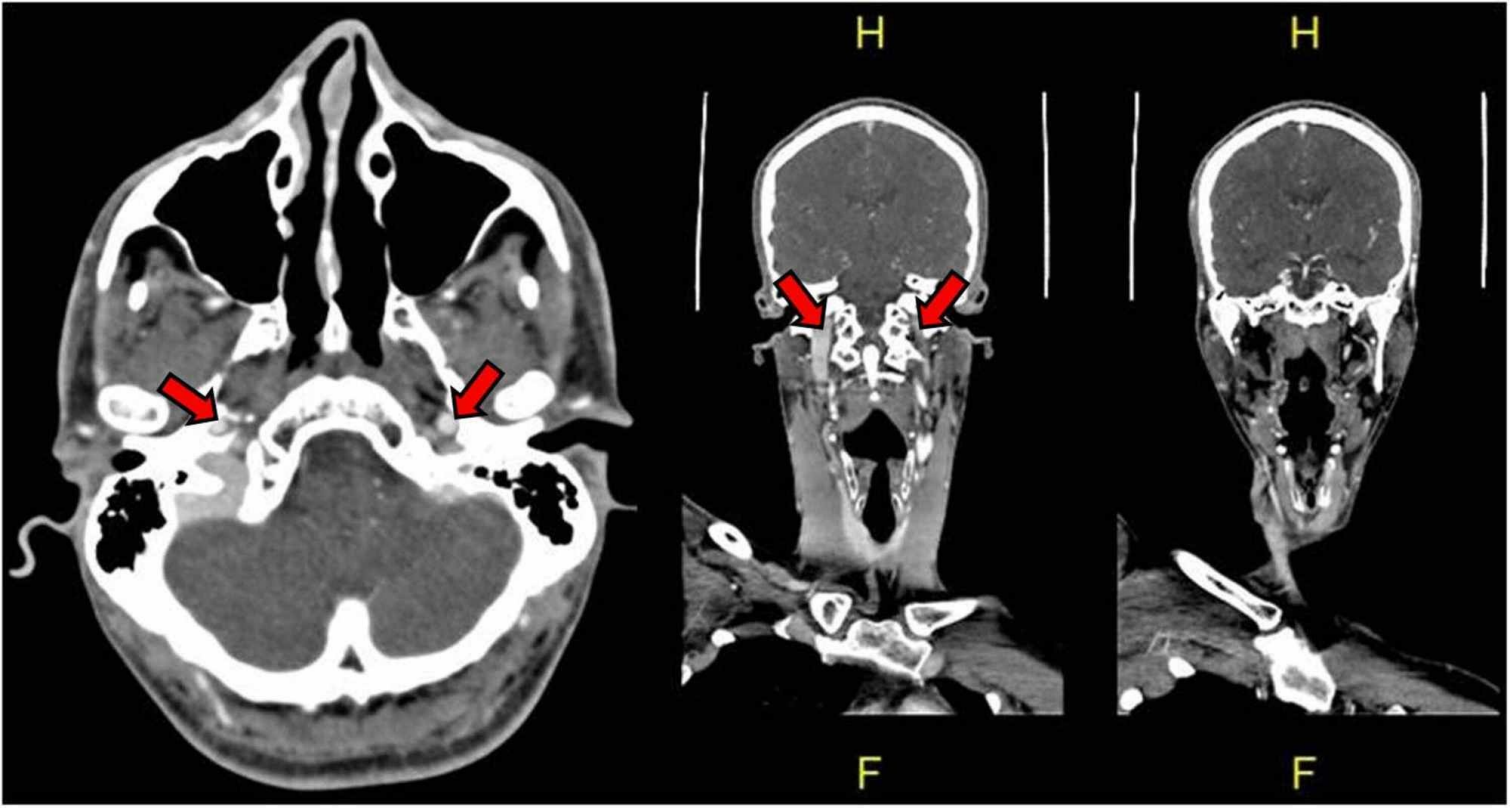
Computed tomography angiogram (CTA) of the vertebral arteries CTA reveals that the distal bilateral vertebral arteries (VA) are thrombosed to the middle of the basilar artery (BA), sparing the left anterior inferior cerebellar artery (AICA) and other parts of the circle of Willis including both posterior communicating arteries.

**Figure 3:**
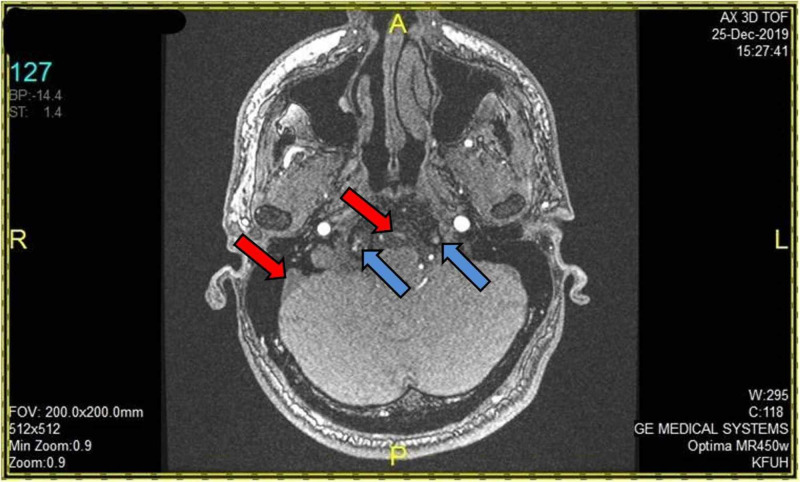
Magnetic resonance angiogram (MRA) of the brain MRA shows acute multiple pontine and right cerebellar peduncle infarctions. Four-vessel cerebral angiography shows a well-organized basilar thrombus with no opacification of the vertebral arteries (VAs) distal to the level of both posterior inferior cerebellar arteries (PICAs) up to the mid-basilar artery, but normal flow in the left anterior inferior cerebellar artery (AICA) as well as the rest of the cerebral vasculature.

**Figure 4:**
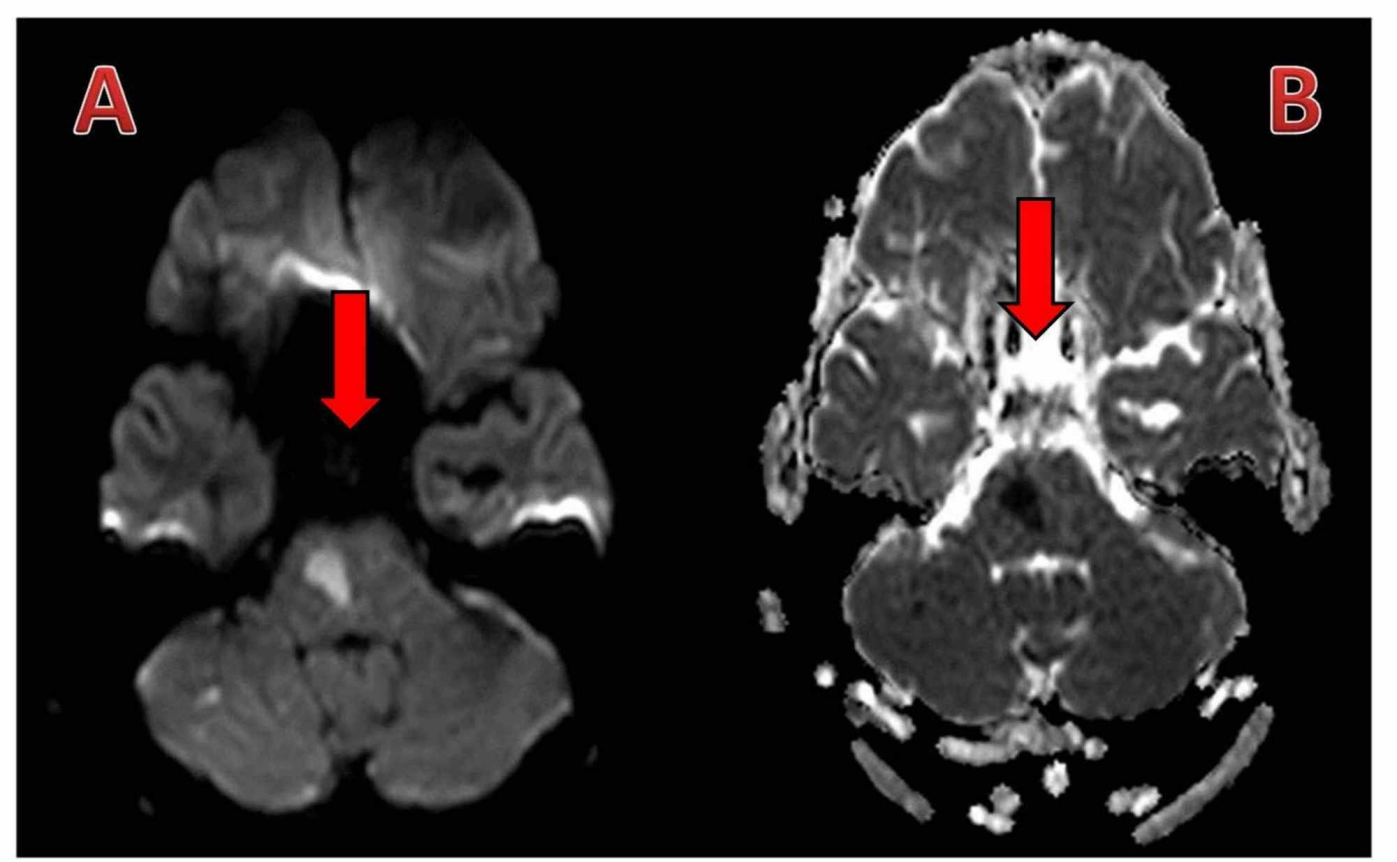
Diffusion weighted imaging (DWI) and apparent diffusion coefficient (ADC) of the brain (A) The acute lesion is a region of hypointensity in the paramedian pontine region. (B) DWI shows the relieved lesion a region of hyperintensity in the paramedian pontine region.

Initial laboratory results were unremarkable. An echo-cardiogram was normal. Computed tomography (CT) scan of the brain revealed pontine hypodensity (Figure 1). CT angiogram (CTA) showed filling defect of both VAs distally till the middle of the basilar artery (BA), sparing the left anterior inferior cerebellar artery (AICA) and other parts of the circle of Willis (Figure 2). Magnetic resonance angiogram (MRA) of the brain showed acute multiple pontine and right cerebellar peduncle infarctions. Four-vessel cerebral angiography showed a well-organized basilar thrombus with no opacification of the VAs distal to the level of both posterior inferior cerebellar arteries (PICA) up to the mid BA, but normal flow in the left AICA and the remaining cerebral vasculature (Figure 3). Diffusion weighted imaging (DWI) and apparent diffusion coefficient (ADC) showed paramedian pontine stroke (Figure 4). The diagnosis of acute VAD was established.

The patient was admitted to the neurocritical care unit. Antiplatelets including clopidogrel 75 mg, and acetylsalicylic acid 100 mg were administered immediately, along with atorvastatin 40 mg and subcutaneous enoxaparin 40 mg daily. Daily intensive physiotherapy was initiated as well. Three months later, the patient recovered completely and returned to his baseline. He was advised to refrain from chiropractic manipulation.

## Discussion

Arterial dissections contribute for 2% of all ischemic strokes. However, in those subjects younger than 45 years, they account for 20% of ischemic stroke presentations [2]. Although no clear gender predominance, women tend to present with VAD five years earlier than men [8]. Arterial dissection can result in adventitial or intimal tears leading to mural hematoma formation that compromises blood flow by narrowing or occluding the vessel. Neurological sequences following VAD can vary from mild neck or occipital pain to catastrophic stroke of the posterior circulation and coma [9].

Patients who are hypertensive, smokers, use oral contraceptives, have connective tissue diseases such as Marfan syndrome and Ehlers-Danlos or have migraines have a higher risk of developing sVAD in association with traumatic events such as chiropractic manipulation, falls, violent coughing, athletic activities, or nose-blowing [6]. In our patient, neck manipulation was determined to be the cause of the sVAD after exclusion of all other causes.

Albedah et al. reported that chiropractic therapy is not very popular among the Saudi population with prevalence of 4.1%. Furthermore, only 11% were performed by medical practitioners while the rest were performed by traditional healers [7]. Hence, chiropractic therapy can be extremely hazardous especially if performed without a medical background or knowledge of the neck anatomy. It is estimated that 1 in 20,000 spinal manipulations results in a vertebral artery dissection. Nonetheless, the exact incidence of this complication is unknown [10]. A thorough search of the literature did not yield any similar cases in Saudi Arabia. Since there is an under reporting of vascular dissection secondary to chiropractic manipulation in the region, we cannot estimate the true impact of such practice as a potential cause of stroke in the Saudi population.

Different neurological findings can accompany sVAD such as vertigo, nystagmus, dysarthria, absent gag reflex with difficulty swallowing, and hemiparesis which are the most commonly reported symptoms [11].

Cranial MRI/MRA is a noninvasive diagnostic tool with a sensitivity of 60% for cerebral changes following arterial dissections. A recent study indicated that CTA is of 100% sensitivity and 98% specificity for the diagnosis of VAD. Conventional four-vessel angiography is the gold standard for diagnosis of VAD [12].

Management of VSD includes several aspects focusing on antithrombotic therapy. Heparin infusion intravenously can be initiated upon presentation followed by oral anticoagulants or antiplatelets for the following three months. Although bleeding is considered the main side effect of these regimens, a recent Cochrane review found no increased risk of ischemic or intracranial events following either regimen [13].

The prognosis of sVAD is generally good, but significant neurologic and cognitive deficits, massive strokes and deaths occur in 4% of patients [14].

## Conclusions

In summary, sVAD should be suspected as cause of stroke in young people. Chiropractic manipulation is a known risk factor for sVAD. Although under reported in Saudi Arabia, it should be investigated as a potential cause; especially that it is mostly performed by people without medical background and hence further potentiating the hazardous effects.
